# Should single embryo transfer be used in patients with any kind of infertility factor? Preliminary outcomes

**DOI:** 10.5935/1518-0557.20190006

**Published:** 2019

**Authors:** Pedro AA Monteleone, Paula GMF Petersen, Pedro FM Peregrino, Juliana Miorin, Alecsandra P Gomes, Mariana G Fujii, Hamilton de Martin, Tatiana CS Bonetti, Sergio P Gonçalves

**Affiliations:** 1 Centro de Reprodução Humana Monteleone, São Paulo -Brazil; 2 Disciplina de Ginecologia - Departamento de Obstetrícia e Ginecologia. Faculdade de Medicina da Universidade de São Paulo, São Paulo -Brazil; 3 Departamento de Ginecologia. Escola Paulista de Medicina da Universidade Federal de São Paulo, São Paulo -Brazil

**Keywords:** Single embryo transfer, infertility factor, clinical pregnancy rate

## Abstract

**Objective:**

Multiple embryos have been transferred to compensate for low implantation rates, which in turn, increase the likelihood of multiple pregnancies. Despite the publication of clinical guidelines and a reduction in the number of embryos transferred, double embryo transfer still is the most common practice. There is no clear evidence of who should receive the single embryo transfer (SET), and it is more commonly indicated for patients of good prognosis. However, it is not clear how much the presence of other infertility factors can affect the SET prognosis. The aim of this study was to evaluate differences in clinical pregnancy rates (CPR) of frozen-thawed SET cycles for women presenting with different infertility factors.

**Methods:**

Retrospective cohort study evaluating 305 frozen-thawed SET cycles performed in the last 10 years in a private IVF center. We included patients undergoing ovarian stimulation cycles, using ejaculated sperm and a frozen-thawed ET. Embryos were routinely vitrified and warmed up, and the blastocysts were transferred after endometrium preparation. The cycles were categorized according to the infertility factor classified by the Society for Assisted Reproductive Technologies (SART) as anatomic female factor (n=55), endocrine female factor (n=26), endometriosis (n=37), male factor (n=60), ovarian insufficiency (n=26), unexplained (n=24), multiple factors (n=45) and other (n=32). CPR were compared between the groups and the multivariate analysis was performed to evaluate the association of each infertility factor and the CPR, adjusted for confounders.

**Results:**

The women varied in age from 18 to 44 years (35.9±3.8), presented Body Mass Index of 22.4±3.1kg/m^2^, baseline serum FSH of 7.4±8.3 IU/ml, and had a mean of 11.0±8.4 MII oocytes recovered and 6.4±5.3 embryos cryopreserved. The CPR, according to infertility factors were: anatomic female factor (25.9%), endocrine female factor (30.8%), endometriosis (27.8%), male factor (20.7%), ovarian insufficiency (21.7%), unexplained (9.5%), multiple factors (17.1%) and other (20.7%). Multivariate analysis did not show significant association of infertility factors and CPR adjusted for confounders.

**Conclusions:**

Patients presenting different infertility factors seem to have a satisfactory CPR for a SET cycle, except those with unexplained infertility. This is a preliminary outcome and the number of patients by category is small; in addition, the retrospective characteristics of the study are its limitations. Overall, our findings suggest that patients presenting any infertility factor, except unexplained infertility, are suitable to receive a SET with satisfactory outcomes.

## INTRODUCTION

Historically, multiple embryos have been transferred in *in vitro* fertilization (IVF) cycles, in an attempt to compensate for the low implantation rates and increase the success of treatment. However, this approach increases the likelihood of multiple pregnancies, which is the main complication of IVF. The publication of clinical guidelines on the number of embryos to transfer and indications of a reduction in the number of embryos transferred (Harbottle *et al*., 2015; ASRM/SART, 2017) prompted a decrease in the transfer of three or more embryos, and increase in the transfer of one or two embryos, but the double embryo transfer is the most common practice yet (Kissin *et al*., 2015).

Ideally, the goal of assisted reproduction techniques (ART) is to achieve a singleton gestation, and a single embryo transfer (SET) is the most effective tool for it (ESHRE Guideline Group on Good Practice in IVF, 2016; ASRM/SART, 2017). On the other hand, low twinning risk is reduced at the expense of declining pregnancy rates in the first cycle, and a need for more embryo transfers to get the same success rate, a potential delay in treatment success, potentially higher treatment costs, together with patient's autonomy to choose placement of more than one embryo tend to result in double embryo transfers done more often (Gleicher & Barad, 2006). Thus, the wide application of SET raises questions, as there is no clear evidence for whom SET should be used. The American Society for Reproductive Medicine encourages individual programs to use their own data regarding patients’ characteristics and the number of embryos transferred, aiming to maintain pregnancy rates and minimizing multiple pregnancies (ASRM/SART, 2017).

Good prognosis patients are more indicated to receive SET with satisfactory clinical outcomes, such as those under the age of 38 and patients at any age transferring an euploid embryo evaluated by preimplantational genetic test for aneuploidy (PGT-A) (ASRM/SART, 2017). A previous study demonstrated that elective SET employed in women younger than 38 years of age in U.S. clinics have decreased multiple pregnancy rates with no impact on cumulative live-birth rates (Mancuso *et al*., 2016). Our group demonstrated that the accumulated outcome of two sequential SET is similar to DET in good prognosis patients (Monteleone *et al*., 2018), and the transfer of two embryos after a SET failure did not have advantages compared to a second SET (Monteleone *et al*., 2016). In addition, the single euploid blastocyst transfer prompts the same clinical outcome of two untested blastocysts, while reducing the risk of multiple pregnancies in women of 42 years or younger (Forman *et al*., 2013). Apart from those characteristics, expectation of one or more high quality embryos available for cryopreservation, or the availability of vitrified high-quality blastocysts for frozen-thawed transfers are also favorable criteria to SET (Richter et al., 2016).

The exact profile of women who are favorable to receive a SET is not well defined, we aimed to evaluate the clinical outcomes of IVF cycles for women who had vitrified embryos and were undergoing frozen-thawed SET because of infertility factors. 

## MATERIAL AND METHODS

This is a preliminary retrospective cohort study evaluating 305 frozen-thawed SET cycles performed in the last 10 years in a private IVF center in Brazil. All procedures in this study are part of the routine care in the assisted reproductive center, and written informed consent was obtained from all patients before treatment. Patients consented to the treatment procedures and to the retrospective data used in the scientific publications.

The database included 1449 frozen-thawed cycles between 2008 and 2017, which were potentially eligible for this study. Then, we deemed eligible for this study if the patient received a frozen-thawed SET. We excluded cycles in which testicular or epididymal sperm were used. The final number of cycles included and analyzed in our study was 305 frozen-thawed SET.

The patients underwent routine ovarian stimulation and oocyte pickup according to the medical criteria for such. The oocytes were fertilized by ICSI, using ejaculated sperm, with or without a fresh embryo transfer according with clinical conditions. Extra embryos for patients who received a fresh transfer, or all embryos for patients who did not receive a fresh transfer, were vitrified for future frozen-thawed transfers. For the frozen-thawed embryo transfers, endometrial preparation was conducted with 100 µg of estradiol valerate (Estradot, Novartis, Switzerland) for 14 days plus 600 µg of vaginal micronized progesterone (Utrogestan, Farmoquimica, Brazil) 5 days before the transfer. The embryos were warmed, evaluated for survival and morphology, and transferred at a blastocyst stage 5 days after progesterone was started. The embryo survival rate after warming was 88.1% and a top-quality blastocyst was preferentially transferred when available.

Frozen-thawed transfers were categorized according to the infertility factor by using the classification established by the Society for Assisted Reproductive Technologies (SART), such as anatomic female factor (n=55), endocrine female factor (n=26), endometriosis (n=37), male factor (n=60), ovarian insufficiency (n=26), unexplained (n=24), multiple factors (n=45) and other (n=32).

### Data analysis

Data was obtained from a clinical report form and plotted for this study. Clinical pregnancy was defined by the presence of a gestational sac with a heartbeat 2 weeks after confirmation of a biochemical pregnancy (serum beta-hCG measurement). The clinical pregnancy rate was calculated as the number of patients presenting a clinical pregnancy divided by the number of patients with embryos transferred.

Analyses were performed using the SPSS V.18 (IBM SPSS Software, USA). The patient demographic data were evaluated using descriptive statistics, which included information on the means, standard deviations and frequencies. The ANOVA was used to compare continuous variables, and the Pearson’s chi-squared of Fisher exact test were used to compare frequencies as appropriated. Regression analyses were used to evaluate the association between the variables, and a multivariate logistic regression analysis was performed to evaluate the association of each infertility factor and clinical pregnancy rate (CPR) adjusted for confounders. The results were reported as odds ratios and *p*-values. We considered *p*-values ≤0.05 to be statistically significant.

## RESULTS

As for the patients included in the study, 135 had a fresh embryo transfer with no pregnancy and the second frozen-thawed SET was evaluated in this study. One-hundred and fourteen had all embryos cryopreserved and the first frozen-thawed SET was evaluated. Table 1 describes the demographic characteristics of the patients included in the study.

**Table 1:** Demographic characteristics of patients included in the study

	Minimum	Maximum	Mean	Std. Deviation
Female age (years)	18	44	35.9	3.8
Male age (years)	27	64	37.3	5.1
BMI (Kg/m^2^)	16	36	22.4	3.1
Infertility duration (years)	0	14	2.6	2.2
Basal FSH measurement (IU/mL)	0.2	87	7.4	8.3
Number of oocytes collected	1	60	11.0	8.4
Number of cryopreserved embryos	1	25	6.4	5.3

The women’s characteristics according to infertility factors are presented on Table 2. As expected, the women classified at ovarian insufficiency category are older than the others with higher basal FSH, had lower numbers of MII oocytes collected and embryos cryopreserved. Except those, the other characteristics were similar between the groups.

**Table 2:** Demographic characteristics of patients included in the study according to infertility factors

	Anatomic female factor	Endocrine female factor	Endometriosis	Male Factor	Ovarian insufficiency	Unexplained	Multiple Factors	Other	ANOVA
n	55	26	37	60	26	32	45	32	
Female age (years)	35.6±3.0	33.9±3.4	35.7±3.3	35.2±3.7	39.8±3.1	36.1±3.6	36.5±4.0	36.0±4.8	<0.001
Male age (years)	36.5±4.0	36.0±4.3	36.9±4.3	37.7±5.7	38.6±4.4	37.4±4.4	38.0±6.7	37.6±5.2	0.517
BMI (Kg/m^2^)	22.3±2.7	22.7±3.7	22.5±2.9	23.3±3.4	22.7±2.6	22.0±3.5	22.2±2.8	22.8±3.7	0.977
Infertility time (years)	2.2±2.2	2.3±1.7	3.3±2.5	2.7±2.4	2.5±2.0	2.8±2.5	2.4±1.8	2.4±1.7	0.492
Basal FSH measurement (IU/mL)	8.0±11.4	5.4±2.3	8.2±5.9	6.8±4.4	12.0± 19.5	6.3±2.4	7.6±5.0	5.4±2.6	0.097
Number of oocytes collected	11.5±6.2	18.5±11.8	8.7±6.7	10.8±6.5	4.3±5.1	9.7±5.2	11.2±10.8	13.3±9.1	<0.001
Number of cryopreserved embryos	7.0±5.0	10.8±5.6	4.9±4.4	6.7±5.1	2.2±2.7	5.8±4.9	7.0±6.4	5.8±4.2	<0.001

**Figure 1:**
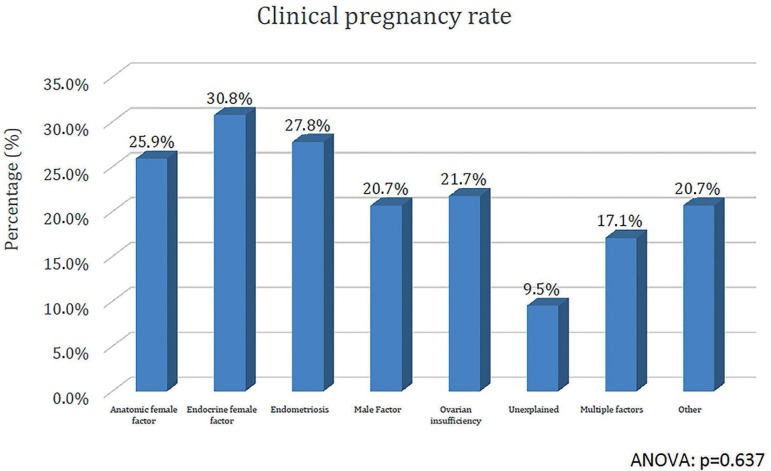
Clinical pregnancy rates according to infertility factor in Frozen-thawed single embryo transfers

CPR after a frozen-thawed SET for each infertility factor is demonstrated in Figure 1. The CPR for patients with different infertility factors seem to be satisfactory for a SET, except for the unexplained infertility that had a very low CPR.

A multivariate logistic regression model was built and adjusted for following confounders based on differences found in univariate analysis (women age and number of embryos cryopreserved) and conditions that could cause biases to the outcomes as if embryos had been evaluated by preimplantational genetic test for aneuploidy or not, and if the patient had a previous fresh embryo transfer or had all embryos cryopreserved. The outcomes did not show any significant association between CPR and infertility factors, such as: anatomic female factor (OR: 1.4; *p*=0.409), endocrine female factor (OR: 1.4; *p*=0.509), endometriosis (OR: 1.7; *p*=0.254), male factor (OR: 1.0; *p*=0.910), ovarian insufficiency (OR: 0.7; *p*=0.720), unexplained (OR: 0.2; *p*=0.117), multiple factors (OR: 0.7; *p*=0.526) and other (OR: 0.7; *p*=0.597). We can note that despite being non-significant, the OR value suggests a decreased likelihood of clinical pregnancy when unexplained infertility is present, adjusted for confounder factors.

## DISCUSSION

IVF success is defined as a singleton pregnancy resulting in a healthy singleton baby born at term (Min *et al*., 2004). While studies have demonstrated for more than a decade that the most effective tool for prevention of twin pregnancies after IVF is SET, and that the cumulative outcomes have comparable live birth rates and diminished multiple gestations (ESHRE Campus Course Report, 2001; Luke *et al*., 2015; ASRM/SART, 2017; Monteleone *et al.,* 2018), there still is some resistance to using SET in general, and questions regarding in which patient it would be most effective (van Peperstraten *et al*., 2008). This preliminary study evaluated frozen-thawed SET for women presenting different infertility factors and demonstrated a tendency of unexplained infertility has the worst clinical outcomes.

Luke *et al*. (2015) developed a study comparing SET and DET, and evaluated the outcomes according to four infertility factors (male factor, ovulation disorder, diminished ovarian reserve and unexplained) and found no differences. Luke *et al*. (2015) included a higher number of patients compared to our preliminary study and evaluated fresh cycles, finding no differences, while we had a tendency to have lower CPR in unexplained infertility of frozen-thawed transfers.

Differences in study design can justify diverse outcomes. Moreover, our study was conducted in an unselected group of patients (i.e. irrespective of the woman’s age or embryo quality) but patients included were those who had cryopreserved embryos and were undergoing a frozen-thawed ET, which is a favorable condition for SET.

Selecting couples suitable for SET is an essential step for the success of the technique. Unexplained infertility is a particular situation in which we do not know the real infertility factor and a number of conditions can be associated, as endometrial dysfunction (Dorostghoal *et al*., 2018; Petousis *et al*., 2018) and autoimmunity (Motak-Pochrzest & Malinowski, 2018). We observed a numerically lower CPR for women presenting unexplained infertility, for that, patients should be extensively evaluated in order to determine the factor associated to infertility and correct it before performing an IVF cycle with SET.

This is a preliminary retrospective study with a small number of cycles in each category, and the outcomes did not show statistical significance. Hence, the findings should be interpreted with caution. On the other hand, the CPR indicates important differences in the subgroup of unexplained infertility and this profile of patients may not be suitable for SET. We built a regression model considering confounding variables as the woman’s age and the number of embryos cryopreserved, preimplantational genetic test for aneuploidy and whether the patient had a previous embryo transfer, since those are conditions that establish a good prognosis for a patient, and we had the same outcomes even after adjustments. The subgroup of unexplained infertility had a very low OR, indicating lower pregnancy likelihood, although not significant. These findings can be considered in the clinical routine when indicating an SET cycle, especially when the patients do not have a known infertility factor.

In short, our study suggests that for couples presenting unexplained infertility, even when a patient has a good prognosis (age lower than 38, preimplantational genetic test for aneuploidy, extra embryos cryopreserved), SET should be considered with caution since the outcomes may be not satisfactory.
